# Visualization of Sex Identification in Red‐Crowned Crane ( *Grus japonensis* ) via Recombinase‐Aided Amplification Combined With *Pyrococcus furiosus* Argonaute Assay

**DOI:** 10.1002/ece3.71780

**Published:** 2025-07-21

**Authors:** Shenluan Tan, Tongtong Zhan, Fanwen Zeng, Xuanjiao Chen, Tanzipeng Chen, Li Li, Hengxi Wei, Shouquan Zhang, Kejing Zuo

**Affiliations:** ^1^ State Key Laboratory of Swine and Poultry Breeding Industry, National Engineering Research Center for Breeding Swine Industry, Guangdong Provincial Key Lab of Agro‐Animal Genomics and Molecular Breeding, College of Animal Science South China Agricultural University Guangzhou China; ^2^ Beijing Zoo, Beijing Key Laboratory of Captive Wildlife Technologies Beijing China; ^3^ Guangzhou Zoo & Guangzhou Wildlife Research Center Guangzhou China

**Keywords:** recombinase‐aided amplification‐ *Pyrococcus furiosus* Argonaute (RAA‐*Pf*Ago), red‐crowned crane, sex identification

## Abstract

The red‐crowned crane ( *Grus japonensis* ), a Class I protected animal in China, inhabits Northeast Asia, including China, Russia, and Japan. As sex‐monomorphic birds, red‐crowned cranes cannot be directly distinguished between females and males through observation. Molecular methods are accurate and stable for sex identification in birds and are widely used in zoos and farms. With the development of isothermal techniques, recombinase‐aided amplification (RAA) has provided novel insights into bird sexing owing to its low equipment dependence and rapid amplification. Advancements in the *Pyrococcus furiosus* Argonaute (*Pf*Ago) biosensor have facilitated clinical detection. In this study, an innovative sex identification system was developed by integrating RAA and *Pf*Ago in red‐crowned cranes. The RAA‐*Pf*Ago system identified both females and males with remarkable accuracy. Via proper design of primers set, gDNA and probe, the RAA‐*Pf*Ago system can complete visual detection, with detection limits between 0.35 ng/μL and 0.035 ng/μL under optimal conditions. The test samples exhibited strong green fluorescence in females, whereas no fluorescence was observed in males under blue light. The results of RAA‐*Pf*Ago in the field were consistent with those obtained using conventional PCR. This study provides a high degree of rapidity, accuracy, and sensitivity for the sex identification of red‐crowned cranes.

## Graphical abstract

This study presents an innovative approach for sex identification in the red‐crowned crane ( *Grus japonensis* ) by combining recombinase‐aided amplification (RAA) with the *Pyrococcus furiosus* Argonaute (*Pf*Ago) biosensor. The RAA‐*Pf*Ago system enables rapid, accurate, and highly sensitive molecular sexing of these sex‐monomorphic birds, with a key advantage being the visual detection of fluorescence. The system achieves a detection limit as low as 0.35 ng/μL and demonstrates consistent performance with traditional PCR methods.

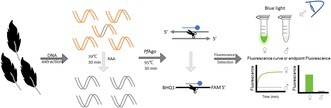

## 1 Introduction

Red‐crowned cranes ( *Grus japonensis* ) are widely distributed across Northeast Asia, including the Yancheng National Nature Reserve and Zhalong National Nature Reserve in China, the middle reaches of the Amur River Basin and the Daurian Steppe of the southern Trans‐Baikal Region in Russia, and Hokkaido, Japan (Dong et al. 2024; Masatomi and Surmach 2018; Smirenski et al. 2018; Su and Zou 2012). The red‐crowned crane belongs to the genus Grus and is well‐known for its vivid red color in its crown. At present, it has been listed as a vulnerable species by the International Union for Conservation of Nature (IUCN); the abundance of mature individuals is approximately 2000–3000, and its population shows a trend of continuous decline (International, B 2021; Su and Zou 2012). Red‐crowned cranes often inhabit wetlands and marine intertidal habitats. The invasion of alien species, such as *Spartina alterniflora* , has altered the ecological structure of nature reserves and negatively impacted the habitats and population structure of red‐crowned cranes (Li et al. 2019; J. Wang et al. 2019). Furthermore, the contraction, pollution, and destruction of wetlands due to residential development and industrial contamination have resulted in a continuous decline in the population of red‐crowned cranes (Li et al. 2025). Some studies have reported that infertility is a common problem in cranes but can be overcome by artificial insemination (Gee 2005). As a result of a combination of anthropogenic pressures and species‐specific biological limitations, red‐crowned cranes are currently facing a critical risk of population decline. Enhancing captive breeding programs and improving reproductive success may help mitigate this trend and contribute to the conservation of the species (Dawei et al. 2024; Lin et al. 2021).

Sex identification is an indispensable step in breeding. However, in many bird species, such as red‐crowned cranes, males and females differ in size, weight, and plumage, which cannot be accurately determined by mere observations (Alonso et al. 2019). Traditional sex identification methods can induce stress in birds and potentially result in mortality (Herrera et al. 2015). Therefore, molecular methods are often used to determine the sex. Sex is determined in birds using ZW chromosomes. In particular, males have two Z chromosomes (ZZ) within avian species, whereas females have one Z and one W chromosome (ZW). This chromosomal difference primarily determines the biological sex of birds and leads to the development of different sexual characteristics and behaviors in males and females (Chue and Smith 2011). The chromodomain helicase DNA‐binding protein 1 (CHD1) gene is highly conserved and on both the Z and W chromosomes. The 2550F/2718R primer set, reported by Fridolfsson and Ellegren, is commonly used in bird sex determination through the polymerase chain reaction (PCR) method, which is performed on the one CHD1‐W fragment in females, and on CHD1‐Z in both sexes (Fridolfsson and Ellegren 1999). However, conventional PCR methods typically require > 3 h for amplification, agarose gel electrophoresis (AGE), and imaging. With the development of isothermal techniques, rapid and facile amplification approaches have become new methods for bird sexing. Recombinase‐aided amplification (RAA) is superior to loop‐mediated isothermal amplification (LAMP) and rolling circle amplification (RCA) in terms of primer design and template selection (Karami et al. 2011; Piepenburg et al. 2006). This technique has high specificity and effectiveness in clinical applications and has been used for sex identification in chicken and pigeons (Lai et al. 2022; Zeng et al. 2024).

The nuclease‐based detection platform, Clustered Regularly Interspaced Short Palindromic Repeats (CRISPR), has recently gained attention as a potent protein for nucleic acid diagnosis, and Cas12‐DETECTOR and Cas13‐SHERLOCK exhibit sensitive and specific detection of pathogens (Li et al. 2023). CRISPR relies on the presence of a specific protospacer‐adjacent motif (PAM) sequence and protospacer‐flanking sequences (PFS) near the target site for recognition and cleavage (Wu et al. 2023). This limits the locations where CRISPR can be used for gene diagnosis and editing, as not every genomic locus contains a suitable PAM sequence.

*Pyrococcus furiosus* Argonaute (*Pf*Ago) is a DNA‐guided nucleic acid endonuclease isolated from thermophilic archaea. It exhibits optimal activity at temperatures ranging from 80°C to 100°C (Swarts et al. 2015). At elevated temperatures, the DNA duplex denatures, allowing *Pf*Ago to target the fragment of interest, guided by a complementary short DNA sequence (guide DNA). *Pf*Ago is less likely to denature under high heat conditions as it is evolutionarily optimized for survival in such environments. This allows *Pf*Ago to perform more effectively at high temperatures. In diagnostics or field applications where conditions may not be controlled, the robustness of *Pf*Ago could be advantageous for tasks such as DNA detection. Without the requirements of PAM and PFS, *Pf*Ago works more flexibly and in a user‐friendly manner than CRISPR. *Pf*Ago‐based systems have been combined with PCR and LCR and have been used for SARS‐CoV‐2 and HPV detection (Wang et al. 2020; L. Wang et al. 2021). The *Pf*Ago system also has been utilized in isothermal amplification assays such as LAMP and RAA, effectively reducing false‐positive rates in pathogen detection. For example, the clinical application of LAMP‐*Pf*Ago in the detection of aquatic pathogens (Pang et al. 2024) and the application of RAA‐*Pf*Ago in the sex identification of flamingos and pigeons has proven the effectiveness, specificity, and sensitivity of the results (Lai et al. 2022; Tan et al. 2024).

**FIGURE 1:**
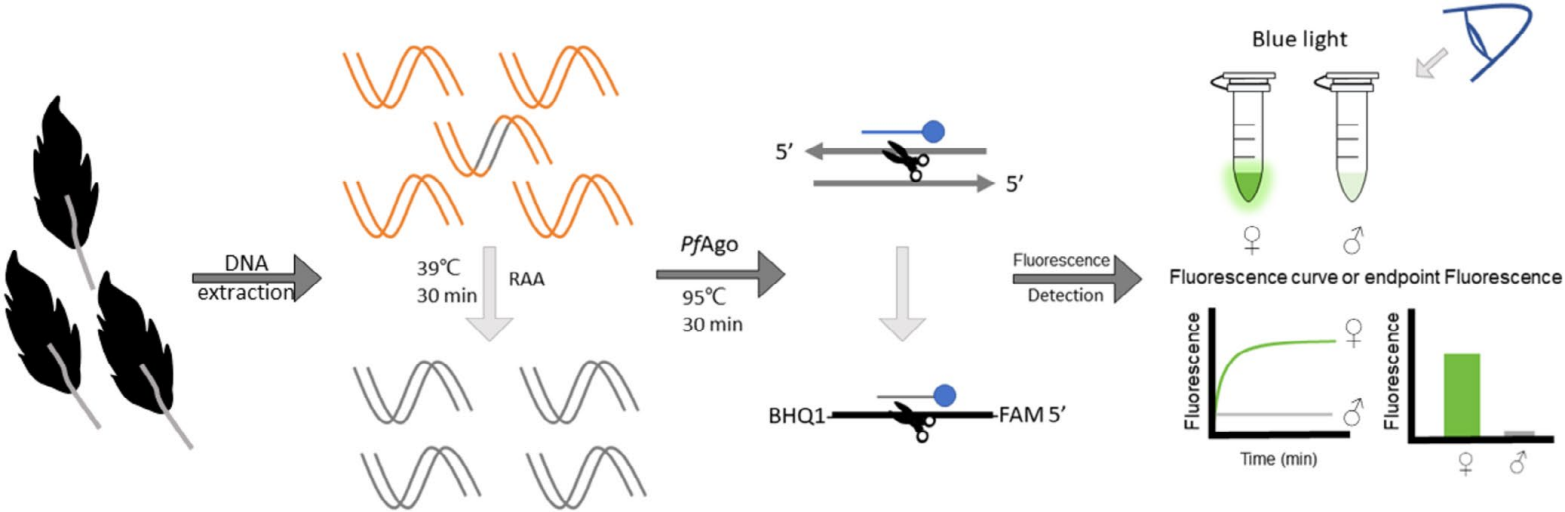
Illustrated scheme of red‐crowned crane‐RAA‐*Pf*Ago sex identification. After DNA extraction from feather samples and their RAA amplification for 30 min at 39°C, the RAA product is added into the *Pf*Ago reaction system and reacted at 95°C for 30 min. Finally, fluorescence is detected done under blue light or a fluorescence detector, and the sample sex is determined.

Previous studies have indicated that RAA has the potential to expand rapidly within 30 min without relying on complex thermal cycling equipment. Furthermore, the *Pf*Ago‐based system exhibited high specificity and sensitivity for onsite detection. This study aimed to establish a visual RAA‐*Pf*Ago method for red‐crowned crane sex identification that is superior to conventional PCR methods in terms of time consumption and operational convenience. The mechanism of red‐crowned crane‐RAA‐*Pf*Ago sex identification is illustrated in Figure 1. DNA was extracted from feather samples and RAA was amplified for the special segment. The *Pf*Ago protein was activated when a 5′‐phosphorylated gDNA was paired with the RAA amplicons; then the *Pf*Ago specifically cleaved phosphodiester bonds between the 10th and 11th bases of the target fragment from the 5′ end. A new 5′‐phosphorylated ssDNA was generated as a gDNA, guiding *Pf*Ago to a secondary cleavage on the probe, which was labeled with FAM as the fluorophore and BHQ‐1 as the quencher. In the absence of the gDNA, FAM and BHQ‐1 were held in close proximity owing to the hairpin‐like structure of the probe and the system could not be fluorescently detected. The newly generated gDNA paired with the probe *Pf*Ago, the probe was cleaved, and FAM was liberated. Fluorescence could then be detected using a fluorescence detector or blue light. This method offers a novel approach for sex identification of red‐crowned cranes and has the potential for application to other crane species.

## 2 Materials and Methods

### 2.1 Sample Collection and DNA Extraction

Feather samples from six red‐crowned cranes of known sex (three males and three females) and 11 red‐crowned cranes of unknown sex were collected and stored in plastic bags from the Guangzhou Zoo, Guangdong Province, China. All experimental animal protocols were reviewed and approved by the Guangzhou Zoo Animal Use and Care Committee (Approval Code: YL2023003). All methods were performed per the relevant regulations and following the recommendations outlined in the ARRIVE guidelines for conducting research. Feathers were excised 2–8 mm from the root end and deposited into a 1.5 mL Eppendorf tube. Total genomic DNA was extracted using the HiPure Tissue & Blood DNA Kit (D3018; Megan, Guangzhou, China), followed by DNA measurement using a Nano‐Drop ND‐2000 spectrophotometer (ThermoFisher Scientific, Waltham, MA, USA); the DNA samples were frozen at −20°C until use.

### 2.2 Primers, gDNA and Probe Design

The primers, gDNA, and probes were designed using Primer Premier 5.0 (PREMIER Biosoft) and synthesized by Sangon Biotech Co. Ltd. (Shanghai, China), as listed in Table 1. The 2550F/2718R primer set was used for PCR as proposed by Fridolfsson and Ellegren (1999). Four pairs of RAA primers were designed according to the design principles for RAA primers. The RAA primers were designed to amplify the CHD‐W segment, and not the CHD‐Z segment, spanning the W (GenBank: EU814909.1) and Z sequences (GenBank: EU814902.1). The gDNA was a synthetic single‐stranded DNA (ssDNA) designed to be complementary to the central region of the RAA amplicon. gDNA was phosphorylated using T4 polynucleotide kinase (New England Biolabs, Ipswich, MA, USA). The probe was engineered to complement the newly generated gDNA and labeled with carboxyfluorescein (FAM) as the fluorophore and Black Hole Quencher‐1 (BHQ‐1) as the quencher.

**TABLE 1:** Primers, gDNA and probe sequences used in this study.

Name	Sequences (5′–3′)
2550F	GTTACTGATTCGTCTACGAGA
2718R	ATTGAAATGATCCAGTGCTTG
RW1‐F	TTGCAGGGGAGGGAATAAGAGTAATGTAAC
RW1‐R	AAGAATTTTGCTGGTAGTAGCCAAGAAGCC
RW2‐F	TTGGCTACTACCAGCAAAATTCTTACCTGA
RW2‐R	TGATTCGTCTACGAGAACGTGGCAACAGAG
RW3‐F	AATTGCAGGGGAGGGAATAAGAGTAATGTA
RW3‐R	TTTCAGGTAAGAATTTTGCTGGTAGTAGCC
RW4‐F	GCAAAATTCTTACCTGAAAGGGAAACTGAC
RW4‐R	TGATTCGTCTACGAGAACGTGGCAACAGAG
gDNA	AAGGCTTCTTGGCTAC
Probe	FAM‐AAGATCAAGGCTTCTTGGCTACTACC‐BHQ1

### 2.3 Conventional PCR Assay

PCR assay was conducted using a 20 μL reaction system containing 2 μL of the DNA solution obtained from feather samples, 10 μL of 2× Dream*Taq* PCR Master Mix polymerase (K1081, ThermoFisher Scientific), 6 μL ddH_2_O, and 1 μL (10 μM) of each primer pair of 2550F/2718R. The thermal cycling parameters were as follows: initial denaturation at 94°C for 3 min; 35 cycles of 94°C for 30 s, 49°C for 45 s, and 72°C for 1 min; followed by a final extension at 72°C for 5 min. PCR amplicons were resolved by electrophoresis on a 1.5% agarose gel and visualized using an ultraviolet transilluminator (Bio‐Rad). To evaluate the sensitivity of the PCR method, 10‐fold serial dilutions of female genomic DNA (35, 3.5, 0.35, 0.035 ng, 3.5, 0.35, and 0.035 pg) were used as templates, and ddH_2_O was used as the negative control. The amplification products were resolved on a 1.5% agarose gel.

### 2.4 RAA Reaction

The RAA reaction was performed using an amplification kit (Zongce Bio‐Sci&Tech Co. Ltd., Hangzhou, China). A total reaction system volume of 50 μL was prepared containing one RAA lyophilized powder, 5 μL DNA template, 25 μL buffer A, 2 μL each of forward and reverse primers (10 μM), 2.5 μL buffer B, and 13.5 μL ddH_2_O, and then incubated at 39°C for 30 min. Four primer sets were selected using the RAA reaction, and the products were subjected to electrophoresis using a 1.5% agarose gel. Moreover, six temperatures (37°C, 38°C, 39°C, 40°C, 41°C, and 42°C) and five durations (10, 15, 20, 30 and 40 min) were tested for the optimal RAA reaction condition using female genomic DNA as templates.

### 2.5 RAA‐ *Pf*Ago Establishment and Evaluation

The 20 μL *Pf*Ago reaction mixture contained 2.0 μL of 10× reaction buffer (150 mM Tris/HCl, 1 M NaCl, and pH 8.0), 2 μL RAA product, 2.66 mM *Pf*Ago (JiaoHong Biotech, Shanghai, China), 150 nM gDNA, 5 mM MgSO_4_, and 500 nM probe, and ddH_2_O was added to a final volume of 20 μL. The reaction was incubated in an ExCycle‐48 Real‐Time PCR Detection System (Glinx Biotech, Shanghai, China) for 30 min at 95°C, and the FAM fluorescence signal was recorded at 30‐s intervals. Genomic DNA templates obtained from six known‐sex (three females and three males) were used to evaluate the specificity of the RAA‐*Pf*Ago sex identification system. To evaluate the sensitivity of the RAA‐*Pf*Ago system, serial dilutions of female genomic DNA identical to those employed in PCR sensitivity evaluation (35, 3.5, 0.35, 0.035 ng, 3.5, 0.35, and 0.035 pg) were used as templates, and ddH_2_O was used as the negative control.

### 2.6 Clinical Sample Detection

Eleven feather samples of unknown sex were collected from a zoo, and genomic DNA was extracted using the previously described protocol. Nucleic acids from the 11 feather samples were detected using RAA‐*Pf*Ago and PCR. PCR detection of red‐crowned cranes was performed using the PCR assay described.

## 3 Results

### 3.1 Optimization of the RAA Conditions

**FIGURE 2:**
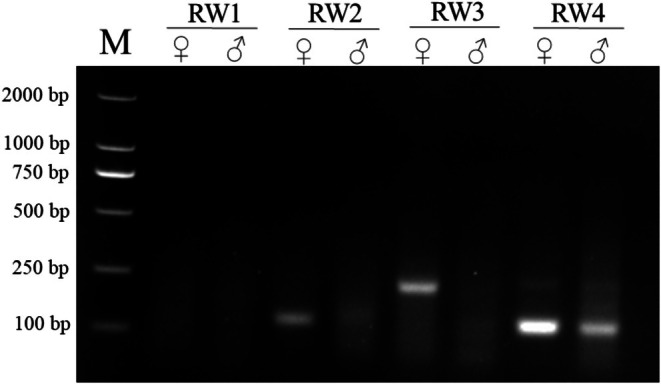
RAA primer selection. The PCR amplicon products were subjected to electrophoresis on a 1.5% agarose gel. M, DL2000 marker. ♀, female; ♂, male.

**FIGURE 3:**
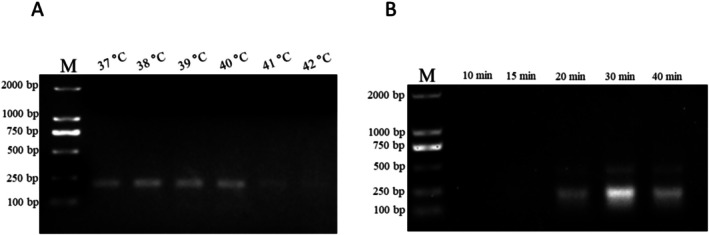
Optimal conditions for RAA. (A) Duration screening for RAA, amplification was performed for varying durations (10, 15, 20, 30, and 40 min) to determine the minimum time required for efficient amplification. (B) Temperature screening for RAA, reactions were conducted at different temperatures (37°C, 38°C, 39°C, 40°C, 41°C and 42°C) to identify the optimal temperature for maximal amplification efficiency. The RAA amplicon products were subjected to electrophoresis on a 1.5% agarose gel. M, DL2000 marker.

Genomic DNA from females and males was used as RAA primer selection templates. After 30 min of reaction at 39°C, RAA products were separated on a 1.5% agarose gel (Figure 2). The RW3 primer set showed specific fragments for CHD‐W but not for CHD‐Z; therefore, RW3 was an effective primer set for RAA. To improve the efficiency of RAA, six temperatures (37°C, 38°C, 39°C, 40°C, 41°C, and 42°C) and five durations (10, 15, 20, 30, and 40 min) were tested. Figure 3 illustrates that the fragment became brighter at 37°C and appeared the brightest at 39°C; there was no indication of the fragment when the temperature exceeded 41°C. In Figure 3B, a faint fragment appeared after 20 min and became brighter after 30 min. Thus, the optimal condition for RAA is achieved at 39°C and 30 min.

### 3.2 Establishment of RAA‐ *Pf*Ago Assay for Sex Identification in Red‐Crowned Cranes

**FIGURE 4:**
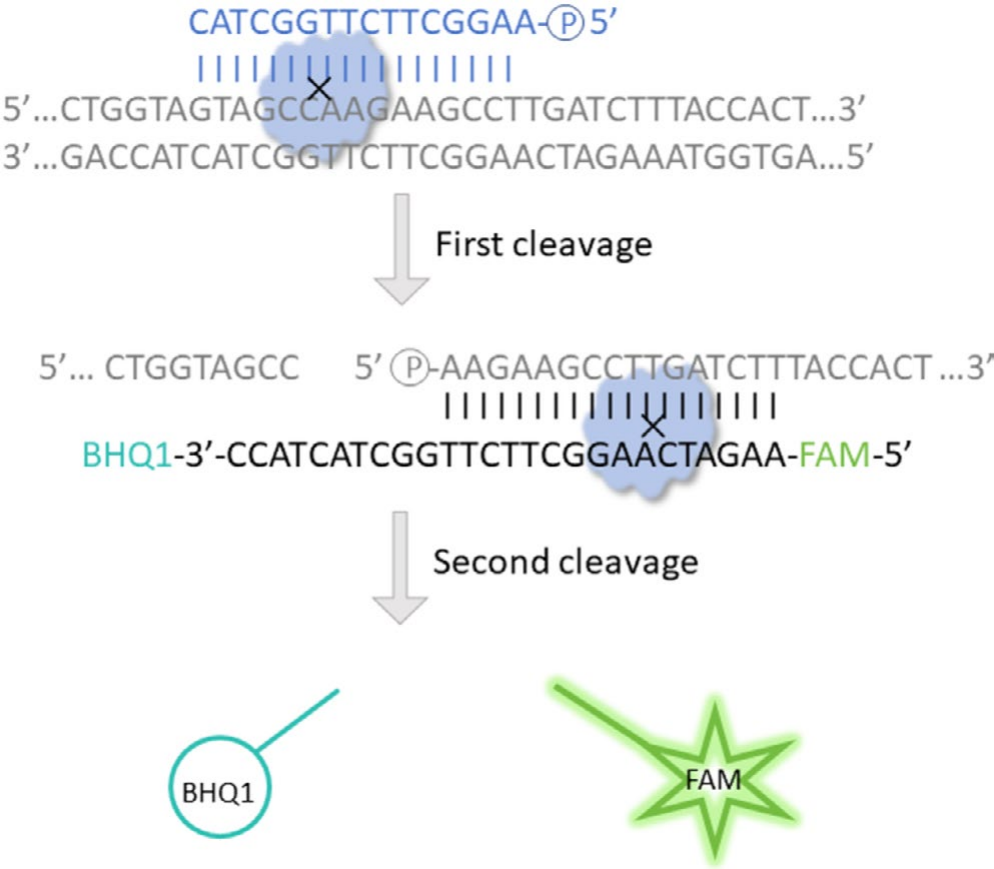
Schematic representation of the *Pf*Ago cleavage reaction in this study. With the guidance of a 5′‐phosphorylated gDNA depicted in blue, *Pf*Ago cleaves phosphodiester bonds between the 10th and 11th bases of the target fragment from the 5′ end, while gDNA is paired with the target DNA depicted in gray. The first cleavage generates a new 5′‐phosphorylated gDNA paired with the probe, which is labeled with FAM as the fluorophore and BHQ‐1 as the quencher, emphasized in cyan and green, respectively. *Pf*Ago cuts the molecular beacon, resulting in the separation of FAM and BHQ‐1, and the fluorescence emitted by the system can be detected.

**FIGURE 5:**
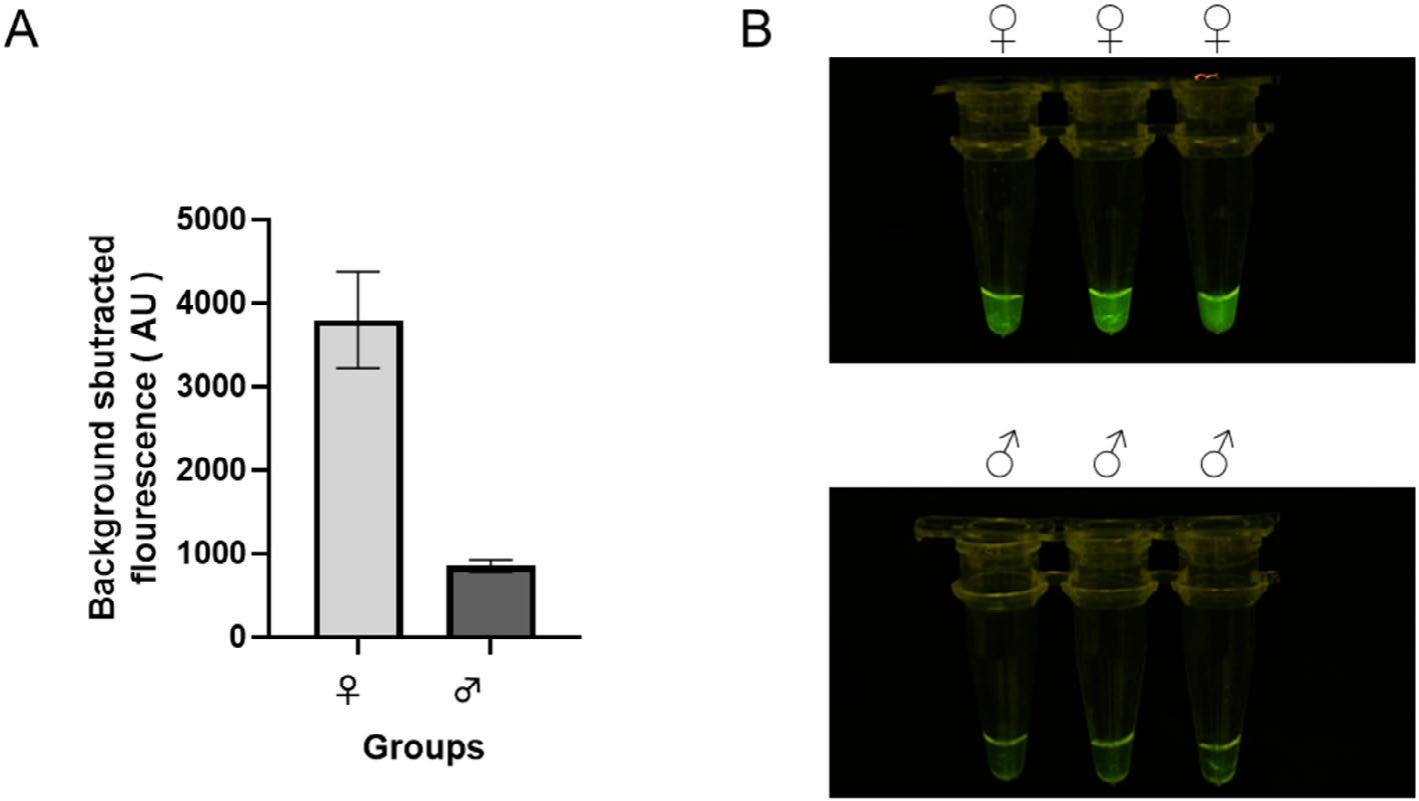
Specificity evaluation of RAA‐*Pf*Ago. (A) Endpoint fluorescence analysis. (B) Observations under blue light.

The gDNA was designed within the amplified region of the RW3 primer set to guide *Pf*Ago in generating new gDNA. The newly generated gDNA‐guided *Pf*Ago completes the second cleavage of the probe. The separation of BHQ‐1 and FAM resulted in the detection of green fluorescence (Figure 4). The ability of the RAA‐*Pf*Ago assay to differentiate between female and male nucleic acids was further validated by incorporating *Pf*Ago, gDNA, and the probe directly into the female or male RAA amplicons. The fluorescence detection results illustrated that the fluorescence values differed between females and males, and the fluorescence value of females was much higher than that of males (Figure 5A). Visual examination under blue light showed strong green fluorescence in females, but no fluorescence signal in males (Figure 5B). The results showed that the RAA‐*Pf*Ago assay was effective in red‐crown crane sexing and highlighted its specificity and precision.

### 3.3 Results of Sensitivity Evaluation

**FIGURE 6:**
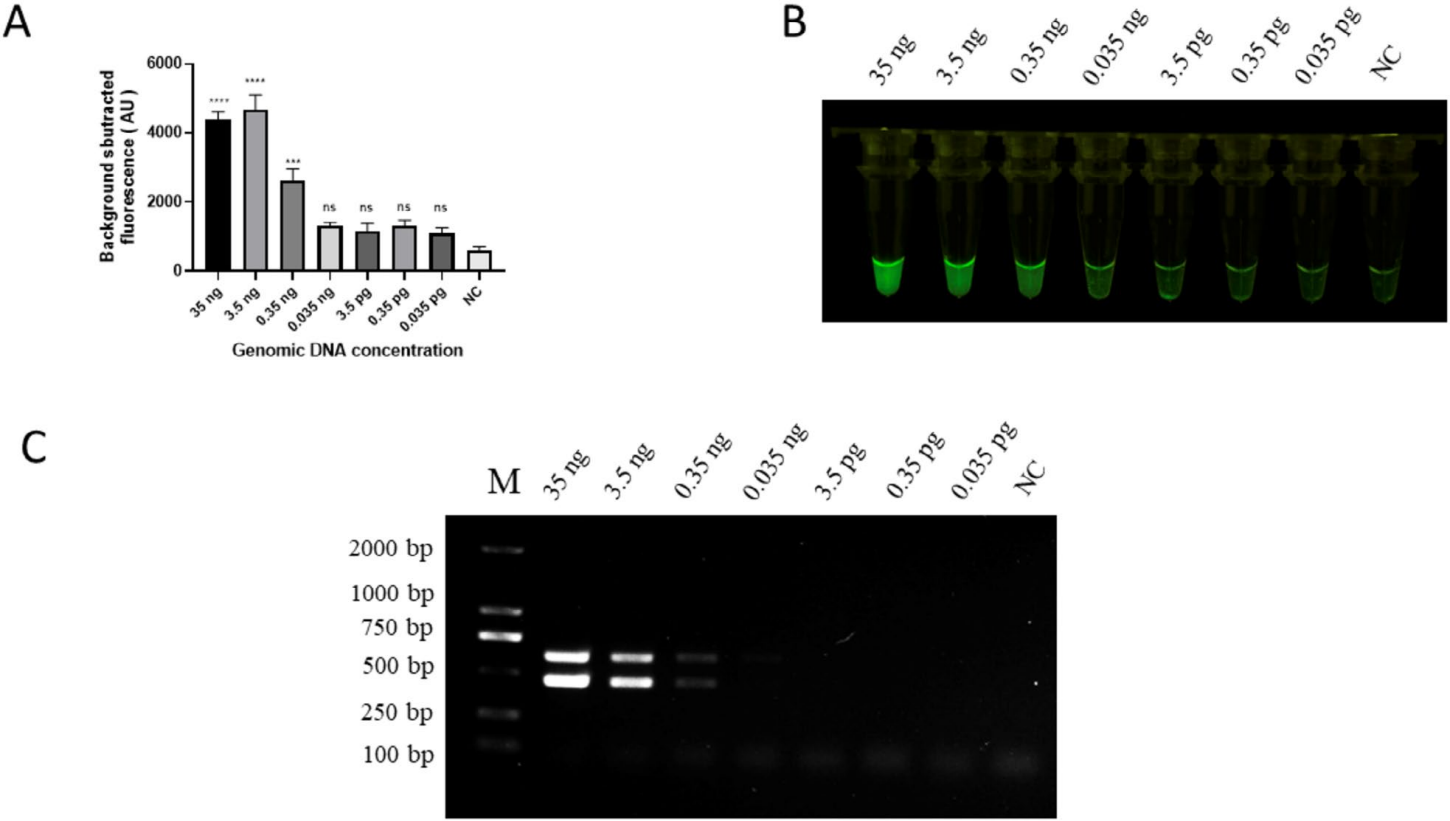
Validation of RAA‐*Pf*Ago and PCR assays sensitivity. (A) Endpoint fluorescence analysis of RAA‐*Pf*Ago sensitivity. (B) Visual detection of RAA‐*Pf*Ago sensitivity products under blue light. (C) Sensitivity of PCR assay evaluate by a 1.5% agarose gel. NC, negative control. Error bars represent SEM; *n* = 3; ***p < 0.001, ****p < 0.0001.

The female genomic DNA sample was diluted 10‐fold (35, 3.5, 0.35, 0.035 ng, 3.5, 0.35, and 0.035 pg) for sensitivity evaluation, and ddH_2_O was used as a negative control. The results showed that the lowest concentration of red‐crowned crane DNA detected by both the RAA‐*Pf*Ago and PCR methods was 0.35 ng/μL (Figure 6A,C). The signal intensity under blue light was noticeably different between 0.35 and 0.035 ng and remained uniform below 0.35 ng (Figure 6B).

### 3.4 Clinical Sample Testing

**FIGURE 7:**
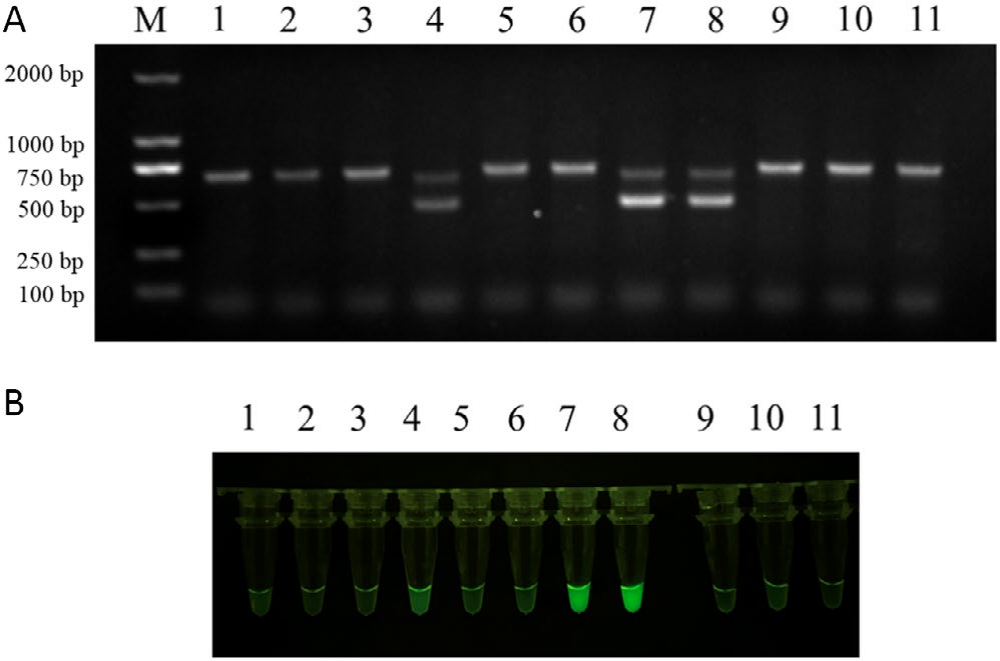
Conventional PCR and RAA‐*Pf*Ago were used for clinical detection. (A) Samples were tested via conventional PCR on a 1.5% agarose gel. (B) Parallel testing was performed using RAA‐*Pf*Ago and visualized under blue illumination.

Eleven red‐crowned crane genomic DNA samples were extracted, as described above. The conventional PCR and RAA‐*Pf*Ago methods were simultaneously tested for clinical sample detection. Samples 4, 7, and 8 were female, while samples 1, 2, 3, 5, 6, 9, 10, and 11 were male (Figure 7A,B). Conventional PCR and RAA‐*Pf*Ago results were consistent, and the RAA‐*Pf*Ago assay showed reliability and validity in red‐crowned crane sex determination, similar to conventional PCR.

## 4 Discussion

The red‐crowned crane, listed as a vulnerable species by the IUCN, continuously faces population constraints driven by extensive habitat degradation, reduced reproductive success, and frequent infertility (Hu et al. 2025; Liu et al. 2020; Zhou et al. 2021). These factors highlight the critical importance of effective conservation strategies, including optimized captive breeding programs, where accurate and timely sex identification—supported by assessment of population structure—plays a fundamental role in the management of red‐crowned cranes. Red‐crowned cranes are sex‐monomorphic birds that cannot be identified based on visual observations alone. Cloacal eversion is widely used in bird sexing; however, the accuracy of this method is related to operator proficiency and can be easily misjudged (Mitchell et al. 2015). Acoustic analysis was reported as an alternative to the classical sexing method, but the species and the developmental stage of birds limit this method (Volodin et al. 2015). PCR‐based molecular identification methods are widely used in farms and zoos for bird sexing. Red‐crowned cranes follow the avian ZW sex‐determination system, with females having two different sex chromosomes (ZW; and males having two identical sex chromosomes [ZZ]). CHD1‐Z and CHD1‐W are located on chromosomes Z and W, respectively. Sex identification was conducted by analyzing the differences in CHD1‐Z and CHD1‐W gene expressions. CHD1 fragments were amplified by PCR using the CHD1‐Z and CHD1‐W genes simultaneously amplified in females, whereas only CHD1‐Z was amplified in males (Griffiths et al. 1996; Masatomi and Masatomi 2018). PCR is widely regarded as a highly accurate and sensitive technique for avian sex determination. However, its reliability depends heavily on the precise design and optimization of primers, as interspecific variation in CHD1 gene sequences among avian taxa can affect amplification efficiency and specificity (Osman et al. 2020). Furthermore, in certain species, the CHD1‐W and CHD1‐Z amplicons exhibit minimal size difference, thereby limiting the resolution of sex identification through conventional AGE, which potentially compromises the accuracy of diagnosis (Ramos et al. 2009). Additionally, PCR‐based methods are limited by their reliance on specialized laboratory equipment and long processing times. Random amplified polymorphic DNA‐PCR (RAPD‐PCR) for crane sex identification requires approximately 10 h (Duan and Fuerst 2001). The advent of isothermal amplification techniques has significantly reduced the amplification duration and mitigated reliance on specific equipment (Zhao et al. 2015).

LAMP, RCA, and RAA are isothermal amplification technologies commonly used for nucleic acid detection. The limitations of LAMP include the stringent requirements for complex primers and reaction conditions, which frequently lead to false‐positive results (Yan et al. 2024). The lack of a template circular DNA limits the use of this technology (Karami et al. 2011). In contrast, RAA does not require an intricate primer design and has high template compatibility. RAA has high efficiency and specificity for amplification. The duration and temperature were optimized in this study; 39°C and 30 min were determined to be the optimal conditions for RAA. The reaction condition in the RAA manual recommends 37°C–39°C for 30 min; however, the temperature and reaction time ranges were expanded to test the effect of temperature and duration time on RAA. Lai et al. attempted 37°C for 25 min of RAA for identifying pigeon sex, similar to this study (Lai et al. 2022). The optimal condition in another study of flamingo sex identification was found to be 40°C for 30 min (Tan et al. 2024). Although the templates were various, the optimal temperature and duration have always been reported around 39°C and 30 min. This may be attributed to the concentration and quality of DNA and primers used in the RAA assay.

The detection platform involves amplification and detection to increase the precision of the results. CRISPR has been combined with RAA to detect numerous pathogens. The restriction of guidance RNA (gRNA) and PAM in CRISPR assays limits their application in testing. Compared with CRISPR, *Pf*Ago works more flexibly in template recognition and gDNA designing (Jiao et al. 2024). *Pf*Ago utilizes a 5′‐phosphorylated 16 nt length DNA but not RNA for guided cleavage of phosphodiester bonds between 10 and 11 from the 5′ end of the target DNA. Furthermore, *Pf*Ago can recognize and cleave target DNA virtually at any site without sequence limitations, such as PAM (Li et al. 2024).

In establishing the red‐crowned crane RAA‐*Pf*Ago assay, gDNA was designed based on the RW3‐F/R primers‐amplified fragment sequence to minimize the possibility of false‐positive results. The RAA product was removed to the *Pf*Ago assay directly and heated at 95°C for 30 min. The enzyme cleaves nearby reporter molecules when a target is detected, producing a visible signal. The results were analyzed by endpoint fluorescence analysis or naked blue light observation. Females showed strong green fluorescence under blue light, but not males. Thus, a visual detection approach was developed for red‐crowned crane sexing in this study, which showed great specificity. To enhance the examination of RAA‐*Pf*Ago, a 10‐fold dilution of female genomic DNA was used for sensitivity testing. These samples were also used for a sensitivity test of the PCR method. According to the results, RAA‐*Pf*Ago could detect at least 0.35 ng/μL of red‐crowned crane female nucleic acid without cross‐reaction. The RAA‐*Pf*Ago method demonstrated high sensitivity and specificity at DNA concentrations of 0.35 ng/μL and above. The PCR method also performed sensitivity at DNA concentrations of 0.35 ng/μL and above, which is identical to the RAA‐*Pf*Ago method. In previous studies, the detection limit of the RAA‐*Pf*Ago method for sex identification in great flamingos was reported to be 0.6 ng/μL (Tan et al. 2024). An RAA‐LFD assay optimized for pigeon sex determination demonstrated a lower detection threshold of 6.3 pg/μL (Lai et al. 2022), while a LAMP‐based method applied to the red‐whiskered bulbul achieved a detection limit of 1 ng/μL (Changtor et al. 2022). Although the sensitivity of the RAA*‐Pf*Ago assay in the present study (0.35 ng/μL) does not represent the lowest value among reported techniques, it falls within a practical and diagnostically relevant range for field‐based applications. In addition to DNA concentration, the integrity of the extracted genomic DNA is a critical factor influencing the performance of the RAA‐*Pf*Ago assay. Although comprehensive assessments of DNA integrity were not conducted in this study, the reproducible and reliable amplification observed across samples suggests that the extracted DNA was of adequate quality to support effective assay performance. Trace or degraded DNA samples could be evaluated for RAA‐*Pf*Ago in future research, potentially expanding its applicability to challenging field samples. For clinical testing of the 11 field samples, the primers 2550F/2718R were used for conventional PCR. The parallel test showed that RAA‐*Pf*Ago showed dependability and precision that were compatible with the PCR results. Compared to the PCR method, the RAA‐*Pf*Ago method is moderately more expensive on a per‐reaction basis (estimated at $6 per test) than the PCR method (estimated at $3 per test), due to the added cost of recombinase proteins, *Pf*Ago enzyme, guide DNAs, and detection reagents (Mahony et al. 2004; Oliveira et al. 2021). However, the increased cost is offset by the method's faster turnaround time, equipment simplicity, and field portability (Yu et al. 2024). These features make it especially valuable for on‐site sex identification in situations where PCR equipment is not available or where rapid results are needed. Currently, the RAA‐*Pf*Ago method was only conducted on red‐crowned cranes due to the limited availability of genomic samples from other crane species. However, according to the NCBI, the CHD‐W gene sequences of several crane species including the Siberian White Crane ( *Grus leucogeranus* ), Hooded Crane ( *Grus monacha* ), Gray Crowned Crane (*Balearica pavonina regulorum*), Common Crane ( *Grus grus* ) and Demoiselle Crane (*Grus virgo*), are identical to that of the red‐crowned crane at the primer, gDNA, and probe binding sites. These species therefore are predicted to be sexed by this method. Future studies should prioritize the validation of the RAA‐*Pf*Ago assay across a broader range of crane taxa.

This study used species‐specific primers, gDNA, probes, and optimal conditions for the red‐crowned crane RAA‐*Pf*Ago sex identification system. This method can achieve complete detection within 1 h without a complex procedure. Under the severe circumstances of animal protection environments, a visual sex identification method can assist practitioners in identifying the sex of red‐crowned cranes more efficiently and accurately, providing a comprehensive understanding of the population structure and breeding conditions of the original inhabitants, newly introduced individuals, and newborns. This research not only reported a new technique for red‐crowned crane sex identification, but also provided new insights into the sexing of other birds.

## Author Contributions

**Shenluan Tan:** writing – review and editing (equal). **Tongtong Zhan:** supervision (equal). **Fanwen Zeng:** data curation (equal). **Xuanjiao Chen:** formal analysis (equal), visualization (equal). **Tanzipeng Chen:** investigation (equal), validation (equal). **Li Li:** software (equal), validation (equal). **Hengxi Wei:** methodology (equal). **Shouquan Zhang:** conceptualization (equal). **Kejing Zuo:** project administration (equal).

## Conflicts of Interest

The authors declare no conflicts of interest.

## Data Availability

All relevant data are included in this paper.

## References

[ece371780-bib-0001] Alonso, J. C. , L. M. Bautista , and J. A. Alonso . 2019. “Sexual Size Dimorphism in the Common Crane, a Monogamous, Plumage‐Monomorphic Bird.” Ornis Fennica 96, no. 4: 194–204.

[ece371780-bib-0002] Changtor, P. , Y. M. Gupta , and N. Yimtragool . 2022. “Optimization and Application of Loop‐Mediated Isothermal Amplification Technique for Sex Identification in Red‐Whiskered Bulbul (*Pycnonotus jocosus*).” Ecology and Evolution 12, no. 10: e9401. 10.1002/ece3.9401. 36225838 PMC9534725

[ece371780-bib-0003] Chue, J. , and C. A. Smith . 2011. “Sex Determination and Sexual Differentiation in the Avian Model.” FEBS Journal 278, no. 7: 1027–1034. 21281451 10.1111/j.1742-4658.2011.08032.x

[ece371780-bib-0004] Dawei, W. , H. Xinyi , C. Hao , C. Guoyuang , C. Weihua , and L. Changhu . 2024. “Breeding Records and the Detection of Nesting Predators of Wild‐Release Red‐Crowned Cranes Into Non‐Breeding Areas of the Yancheng National Nature Reserve, China.” Ecology and Evolution 14, no. 4: e11322. 10.1002/ece3.11322. 38651165 PMC11033623

[ece371780-bib-0005] Dong, W. , K. Tomita , A. Sawada , et al. 2024. “Possible Shifts in the Genetic Diversity of Red‐Crowned Cranes (*Grus japonensis*) in Hokkaido, Japan: Indications of Continental Gene Flow.” Animals 14, no. 11: 1633. 38891680 10.3390/ani14111633 PMC11171382

[ece371780-bib-0006] Duan, W. , and P. Fuerst . 2001. “Isolation of a Sex‐Linked DNA Sequence in Cranes.” Journal of Heredity 92, no. 5: 392–397. 11773245 10.1093/jhered/92.5.392

[ece371780-bib-0007] Fridolfsson, A.‐K. , and H. Ellegren . 1999. “A Simple and Universal Method for Molecular Sexing of Non‐Ratite Birds.” Journal of Avian Biology 30: 116–121.

[ece371780-bib-0008] Gee, G. F. 2005. “Crane Reproductive Physiology and Conservation.” Zoo Biology 2, no. 3: 199–213. 10.1002/zoo.1430020306.

[ece371780-bib-0009] Griffiths, R. , S. Daan , and C. Dijkstra . 1996. “Sex Identification in Birds Using Two CHD Genes.” Proceedings of the Royal Society of London, Series B: Biological Sciences 263, no. 1374: 1251–1256. 10.1098/rspb.1996.0184 8858876

[ece371780-bib-0010] Herrera, A. M. , P. L. Brennan , and M. J. Cohn . 2015. “Development of Avian External Genitalia: Interspecific Differences and Sexual Differentiation of the Male and Female Phallus.” Sexual Development 9, no. 1: 43–52. 25011524 10.1159/000364927

[ece371780-bib-0011] Hu, X. , D. Wu , H. Chen , et al. 2025. “Will Red‐Crowned Cranes Avoid Coastal Wind Farms? A Research Based on Satellite Tracking in Yancheng Coastal Wetland.” Journal of Environmental Management 373: 123508. 10.1016/j.jenvman.2024.123508. 39637497

[ece371780-bib-0012] International, B . 2021. “*Grus japonensis* (Errata Version Published in 2022). The IUCN Red List of Threatened Species 2021.” Retrieved From e.T22692167A213488064.

[ece371780-bib-0013] Jiao, J. , D. Zeng , Y. Wu , C. Li , and T. Mo . 2024. “Programmable and Ultra‐Efficient Argonaute Protein‐Mediated Nucleic Acid Tests: A Review.” International Journal of Biological Macromolecules 278: 134755. 39147338 10.1016/j.ijbiomac.2024.134755

[ece371780-bib-0014] Karami, A. , P. Gill , M. Kalantar Motamedi , and M. Saghafinia . 2011. “A Review of the Current Isothermal Amplification Techniques: Applications, Advantages and Disadvantages.” Journal of Global Infectious Diseases 3, no. 3: 293–302.21887064

[ece371780-bib-0015] Lai, F. Y. , K. C. Chang , C. S. Chang , and P. H. Wang . 2022. “Development of a Rapid Sex Identification Method for Newborn Pigeons Using Recombinase Polymerase Amplification and a Lateral‐Flow Dipstick on Farm.” Animals 12, no. 21: 2969. 10.3390/ani12212969. 36359091 PMC9656852

[ece371780-bib-0016] Li, N. , Z. Wang , L. Xia , et al. 2019. “Effects of Long‐Term Coastal Reclamation on Suitable Habitat and Wintering Population Size of the Endangered Red‐Crowned Crane, *Grus japonensis* .” Hydrobiologia 827: 21–29.

[ece371780-bib-0017] Li, Y. , D. Liao , J. Kou , et al. 2023. “Comparison of CRISPR/Cas and Argonaute for Nucleic Acid Tests.” Trends in Biotechnology 41, no. 5: 595–599. 10.1016/j.tibtech.2022.11.002. 36494308

[ece371780-bib-0018] Li, Y. , L. Zhao , J. Wang , L. Ma , Y. Bai , and F. Feng . 2024. “Argonaute‐Based Nucleic Acid Detection Technology: Advantages, Current Status, Challenges, and Perspectives.” ACS Sensors 9, no. 11: 5665–5682. 10.1021/acssensors.4c01631. 39526595

[ece371780-bib-0019] Li, Y. , Y. Zhuang , J. Dong , et al. 2025. “Red‐Crowned Cranes ( *Grus japonensis* ) Habitat Changes in China From 1980 to 2020: Spatio‐Temporal Distribution.” Journal of Environmental Management 376: 124501. 39954500 10.1016/j.jenvman.2025.124501

[ece371780-bib-0020] Lin, Y.‐f. , P. Xu , W.‐w. Zhang , P. Cui , X.‐m. Li , and C.‐h. Lu . 2021. “Status of Red‐Crowned Cranes (*Grus japonensis*) in Captivity in Zoos and Nature Reserves in China.” Journal of Ecology and Rural Environment 37, no. 5: 668–673. 10.19741/j.issn.1673-4831.2020.0448.

[ece371780-bib-0021] Liu, L. , J. Liao , Y. Wu , and Y. Zhang . 2020. “Breeding Range Shift of the Red‐Crowned Crane ( *Grus japonensis* ) Under Climate Change.” PLoS One 15, no. 3: e0229984. 10.1371/journal.pone.0229984. 32163476 PMC7067427

[ece371780-bib-0022] Mahony, J. B. , A. Petrich , L. Louie , et al. 2004. “Performance and Cost Evaluation of One Commercial and Six In‐House Conventional and Real‐Time Reverse Transcription‐Pcr Assays for Detection of Severe Acute Respiratory Syndrome Coronavirus.” Journal of Clinical Microbiology 42, no. 4: 1471–1476. 10.1128/jcm.42.4.1471-1476.2004. 15070991 PMC387602

[ece371780-bib-0023] Masatomi, H. , and Y. Masatomi . 2018. “Ecology of the Red‐Crowned Crane and Conservation Activities in Japan.” Biodiversity Conservation Using Umbrella Species, 83–105. Springer.

[ece371780-bib-0024] Masatomi, Y. , and S. G. Surmach . 2018. “Distribution of the Red‐Crowned Crane in the World.” Biodiversity Conservation Using Umbrella Species, 73–82. Springer.

[ece371780-bib-0025] Mitchell, C. , P. Cranswick , S. Kharitonov , et al. 2015. “Biometrics of Wild Red‐Breasted Geese *Branta ruficollis* .” Wild 65, no. 65: 154–166.

[ece371780-bib-0026] Oliveira, B. B. , B. Veigas , and P. V. Baptista . 2021. “Isothermal Amplification of Nucleic Acids: The Race for the Next “Gold Standard”.” Frontiers in Sensors 2: 752600.

[ece371780-bib-0027] Osman, M. A. , S. Sugnaseelan , J. M. Panandam , and N. I. Ab Ghani . 2020. “Molecular Sex Identification of Malaysian White‐Nest Swiftlet ( *Aerodramus fuciphagus* Thunberg, 1812).” Ecology and Evolution 10, no. 19: 10440–10448. 10.1002/ece3.6699. 33072271 PMC7548184

[ece371780-bib-0028] Pang, F. , T. Zhang , F. Dai , et al. 2024. “A Handheld Isothermal Fluorescence Detector for Duplex Visualization of Aquatic Pathogens via Enhanced One‐Pot LAMP‐PfAgo Assay.” Biosensors and Bioelectronics 254: 116187. 38518558 10.1016/j.bios.2024.116187

[ece371780-bib-0029] Piepenburg, O. , C. H. Williams , D. L. Stemple , and N. A. Armes . 2006. “DNA Detection Using Recombination Proteins.” PLoS Biology 4, no. 7: e204. 16756388 10.1371/journal.pbio.0040204 PMC1475771

[ece371780-bib-0030] Ramos, P. S. , E. Bastos , R. W. Mannan , and H. Guedes‐Pinto . 2009. “Polymerase Chain Reaction‐Single Strand Conformation Polymorphism Applied to Sex Identification of *Accipiter cooperii* .” Molecular and Cellular Probes 23, no. 2: 115–118. 10.1016/j.mcp.2008.12.002. 19111606

[ece371780-bib-0031] Smirenski, S. M. , E. M. Smirenski , S. G. Surmach , Y. Masatomi , and K. Momose . 2018. Ecology and Conservation of Red‐Crowned Cranes in Russia, 107–128. Biodiversity Conservation Using Umbrella Species: Blakiston's Fish Owl and the Red‐crowned Crane.

[ece371780-bib-0032] Su, L. , and H. Zou . 2012. “Status, Threats and Conservation Needs for the Continental Population of the Red‐Crowned Crane.” Avian Research 3, no. 3: 147–164.

[ece371780-bib-0033] Swarts, D. C. , J. W. Hegge , I. Hinojo , et al. 2015. “Argonaute of the Archaeon *Pyrococcus furiosus* Is a DNA‐Guided Nuclease That Targets Cognate DNA.” Nucleic Acids Research 43, no. 10: 5120–5129. 25925567 10.1093/nar/gkv415 PMC4446448

[ece371780-bib-0034] Tan, S. , F. Zeng , W. Zhong , et al. 2024. “Using Recombinase‐Aid Amplification Combined With *Pyrococcus furiosus* Argonaute for Rapid Sex Identification in Flamingo (Phoenicopteridae).” Animals 15, no. 1: 7. 39794950 10.3390/ani15010007 PMC11718780

[ece371780-bib-0035] Volodin, I. A. , E. V. Volodina , A. V. Klenova , and V. A. Matrosova . 2015. “Gender Identification Using Acoustic Analysis in Birds Without External Sexual Dimorphism.” Avian Research 6: 1–17.

[ece371780-bib-0036] Wang, F. , J. Yang , R. He , et al. 2020. “PfAgo‐Based Detection of SARS‐CoV‐2.” Biosensors & Bioelectronics 177: 112932. 33429204 10.1016/j.bios.2020.112932 PMC7832551

[ece371780-bib-0037] Wang, J. , H. Liu , Y. Li , et al. 2019. “Effects of *Spartina alterniflora* Invasion on Quality of the Red‐Crowned Crane ( *Grus japonensis* ) Wintering Habitat.” Environmental Science and Pollution Research International 26, no. 21: 21546–21555. 10.1007/s11356-019-05408-3. 31127519

[ece371780-bib-0038] Wang, L. , R. He , B. Lv , et al. 2021. “ *Pyrococcus furiosus* Argonaute Coupled With Modified Ligase Chain Reaction for Detection of SARS‐CoV‐2 and HPV.” Talanta 227: 122154. 33714462 10.1016/j.talanta.2021.122154 PMC7875706

[ece371780-bib-0039] Wu, Z. , L. Yu , W. Shi , and J. Ma . 2023. “Argonaute Protein‐Based Nucleic Acid Detection Technology.” Frontiers in Microbiology 14: 1255716. 10.3389/fmicb.2023.1255716. 37744931 PMC10515653

[ece371780-bib-0040] Yan, S. , C. Li , H. Lan , D. Pan , and Y. Wu . 2024. “Comparison of Four Isothermal Amplification Techniques: LAMP, SEA, CPA, and RPA for the Identification of Chicken Adulteration.” Food Control 159: 110302.

[ece371780-bib-0041] Yu, Z. , D. Shi , Y. Dong , et al. 2024. “ *Pyrococcus furiosus* Argonaute Combined With Loop‐Mediated Isothermal Amplification for Rapid, Ultrasensitive, and Visual Detection of Fowl Adenovirus Serotype 4.” Poultry Science 103, no. 7: 103729. 10.1016/j.psj.2024.103729. PMC11066553 38676965

[ece371780-bib-0042] Zeng, F. , W. Zhong , T. Chen , et al. 2024. “Sex Identification of a Multispecies Carinatae Birds by Chicken EE0. 6 Gene Using Real‐Time Recombinase‐Aid Amplification Assay.” Ecology and Evolution 14, no. 11: e70551. 39563704 10.1002/ece3.70551 PMC11575936

[ece371780-bib-0043] Zhao, Y. , F. Chen , Q. Li , L. Wang , and C. Fan . 2015. “Isothermal Amplification of Nucleic Acids.” Chemical Reviews 115, no. 22: 12491–12545. 26551336 10.1021/acs.chemrev.5b00428

[ece371780-bib-0044] Zhou, D. , H. Zhang , X. Zhang , W. Zhang , T. Zhang , and C. Lu . 2021. “Habitat Changes in the Most Important Stopover Sites for the Endangered Red‐Crowned Crane in China: A Large‐Scale Study.” Environmental Science and Pollution Research 28, no. 39: 54719–54727. 34018109 10.1007/s11356-021-14488-z

